# Validity and Reliability of New Agility Test among Elite and Subelite under 14-Soccer Players

**DOI:** 10.1371/journal.pone.0095773

**Published:** 2014-04-21

**Authors:** Younés Hachana, Helmi Chaabène, Ghada Ben Rajeb, Riadh Khlifa, Ridha Aouadi, Karim Chamari, Tim J. Gabbett

**Affiliations:** 1 Research Unit: Analyse et Évaluation des Facteurs Affectant la Performance Sportive, Institut Supérieur du Sport et de l’Éducation Physique, Ksar Said, Tunisia. Université de la Manouba, Tunis, Tunisia; 2 Tunisian Research Laboratory “Sport Performance Optimization,” National Centre of Medicine and Science in Sports (CNMSS), Tunis, Tunisia; 3 School of Exercise Science, Australian Catholic University, Brisbane, Australia; 4 School of Human Movement Studies, the University of Queensland, Brisbane, Australia; 5 Athlete Health and Performance Research Centre, ASPETAR, Qatar Orthopaedic and Sports Medicine Hospital, Doha, Qatar; Groupe IRIS CNRS, UMR8194, Paris Descartes, France

## Abstract

**Background:**

Agility is a determinant component in soccer performance. This study aimed to evaluate the reliability and sensitivity of a “Modified Illinois change of direction test” (MICODT) in ninety-five U-14 soccer players.

**Methods:**

A total of 95 U-14 soccer players (mean ± SD: age: 13.61±1.04 years; body mass: 30.52±4.54 kg; height: 1.57±0.1 m) from a professional and semi-professional soccer academy, participated to this study. Sixty of them took part in reliability analysis and thirty-two in sensitivity analysis.

**Results:**

The intraclass correlation coefficient (ICC) that aims to assess relative reliability of the MICODT was of 0.99, and its standard error of measurement (SEM) for absolute reliability was <5% (1.24%). The MICODT’s capacity to detect change is “good”, it’s SEM (0.10 s) was ≤ SWC (0.33 s). The MICODT is significantly correlated to the Illinois change of direction speed test (ICODT) (r = 0.77; p<0.0001). The ICODT’s MDC_95_ (0.64 s) was twice about the MICODT’s MDC_95_ (0.28 s), indicating that MICODT presents better ability to detect true changes than ICODT. The MICODT provided good sensitivity since elite U-14 soccer players were better than non-elite one on MICODT (p = 0.005; ***d_z_*** = 1.01 [large]). This was supported by an area under the ROC curve of 0.77 (CI 95%, 0.59 to 0.89, p<0.0008). The difference observed in these two groups in ICODT was not statistically significant (p = 0.14; ***d_z_*** = 0.51 [small]), showing poor discriminant ability.

**Conclusion:**

MICODT can be considered as more suitable protocol for assessing agility performance level than ICODT in U-14 soccer players.

## Introduction

Agility is a common term that is used by strength and conditioning practitioners and is often considered the basic element of many sports and activities. A soccer player changes direction every 2–4 seconds and makes 1,200–1,400 changes of direction during a game [1]. Thus agility is a fundamental physical quality for the optimal performance of soccer players [2].

In most research, the term agility has been applied to describe any quick and effective combination of braking, changing direction, and accelerating again while maintaining motor control in either a vertical or horizontal direction in response to a stimulus (i.e., an opposing player’s movements, movement of the ball) [3], [4]. In this context, in order to perform successfully, team sport players need to be good movers in different directions, and often, in confined spaces [5]. Thus, improving the change of direction speed (CODS) of soccer players has become a focus of training programmes and consequently, many studies have been conducted to enhance and assess this athletic quality [6], [7].

It has been well established that fitness testing is crucial for sport performance optimization. It is a feature of most contemporary team sport codes. It quantifies and monitors the discrete training-induced adaptations and effects of training and conditioning program. It is not surprising that a strong interest exists in developing and validating field tests of CODS (e.g., the T-test) [7], the Illinois agility test [8], the 505 test [9], the L-run [10], and the zigzag test [11] in order to allow researchers to effectively measure the specific CODS of team sport athletes. The Illinois CODS test (ICODT) is well accepted as a standard test of change of direction speed in team sports and has been used extensively, particularly in soccer players [12], [13], [14], [15], [16]. The ICODT is a timed task involving straight sprinting and multiple direction changes around obstacles. The generic cues of the ICODT closely replicate the majority of those of the movement pattern of soccer. According to Brughelli et al. [12], the ICODT can be described as a rare CODS test that incorporates as many as 12 changes of direction with a dominant horizontal application of force during the effort. However, the ICODT performance might be heavily influenced by the ability to sprint quickly over short distances [17]. Moreover, the duration of this CODS test is approximately 16–18 seconds, and the total sprinting distance covered is approximately 60 m. However, it is well established that the mean distance and duration of sprints during a match in young soccer players is∼25 m and ∼3s, respectively [18]. Therefore, the ICODT might not represent a sport-specific test for young soccer players. Moreover, this test might be overly strenuous for young players, which might also affect the validity and/or reliability of the test. In order to respond to the need for a more specific CODS test for young soccer athletes, we propose a modified version of the ICODT (i.e., MICODT). In this new version, the same nature of directional changes was maintained, but we reduced the total distance covered (i.e., ∼30 m). Moreover the starting position was modified from laying on the ground in a prone position to a standing position. Indeed, the original starting position was not specific to soccer, as almost all bouts during a match are triggered from a standing position.

Thus, the aims of this study were, firstly to examine the absolute and relative reliability of MICODT and ICODT in U-14 soccer players, and secondly to investigate if a MICODT and ICODT could reveal differences in CODS performance between two U-14 soccer players group of different levels of expertise (i.e., elite and non-elite group).

## Methods

### Experimental Approach to the Problem

Young athletes are widely involved in soccer practice. A soccer match final result is widely recognized to be mainly function of the high speed action performed. Therefore, assessing the change of direction speed of young athletes seems to be extremely needed. To that end, young soccer players involved within the present study performed the ICOD and MICOD tests with a self-administered start in random order and in two different days (24 hours apart). In the first day athletes performed the MICODT and in the second day they performed again the MICODT and the ICODT. All athletes performed three trials of each tests within the two sessions. Recovery period was 3 minutes between trials and 5 minutes between tests (i.e., MICODT and ICODT) to ensure adequate recovery [17]. The best performance was recorded for each player during the two tests. Test procedures were performed in a synthetic surface at the same time of day (i.e., 7–9 p.m.) and in similar environmental conditions (i.e., 24–26°C, 50–60% humidity). The participants were instructed to maintain consistent dietary and sleeping patterns for 48 hours before each session and to refrain from strenuous activity for 24 hours before each session. Before testing, subjects completed a 15-min warm-up, including jogging, lateral displacements, dynamic stretching and jumping.

### Participants

The human subject committee of the local university (i.e., higher institute of sport and physical education Ksar said, Tunis, Tunisia) approved the study in accordance with the 1975 Declaration of Helsinki. Subjects and parental written informed consent was given prior to participation in this investigation. A total of 95 U-14 soccer players (mean ± SD: age: 13.61±1.04 years; body mass: 30.52±4.54 kg; height: 1.57±0.1 m) from a professional and semi-professional soccer academy, took part in this study. Participants trained at least three times per week for two hours per session. All participants had been involved in soccer training regularly for more than four years before the study. All subjects were free from any injury that would prevent maximal effort during performance testing. Moreover, they were frequent participants in experimental studies and were fully familiarized with the test procedures, prior to the onset of the study. For the reliability study of the ICODT and MICODT tests scores, 60 players (out of the 95 subjects) (mean ± SD: age = 13.8±1.5 years; body mass = 33.3±5.2 kg; height = 1.6±0.3 m) took part in this first phase. To investigate the construct validity of the MICODT, a group contained a total of 32 athletes divided to two groups of 16 players based on their expertise level participated to this second phase. Athletes from professional soccer academies (i.e., involved regularly in national level match) were assigned as an elite group (EG) (mean ± SD: age = 13.9±0.7 year; body mass = 34.2±5.2 kg; height = 1.6±0.2 m) whereas those who were from the semi-professional academy (i.e., involved only in local level match) (mean ± SD: age = 13.7±1.4 years; body mass = 33.2±4.3 kg; height = 1.3±0.2 m) were assigned to a sub-elite group (SEG). Non-significant (p>0.05) differences in mean age, height, and body mass were noted among the two groups. Also, there were non-significant (p>0.05) differences in the mean values of the maturation index of the players. This index was calculated using the following equation of Mirwald et al [19]: *Maturity offset = −9.236+0.0002708×(Leg Length×Sitting Height)−0.001663×(Age×Leg Length)+0.007216×(Age×Sitting Height)+0.02292×(mass/Height)* with R = 0.94, R^2^ = 0.891 and the standard error of estimate = 0.592. This is a non-invasive procedure of predicting years from peak height velocity as a measure of offset using anthropometric variables. Negative values were interpreted as pre-peak height velocity and positive values were interpreted as post-peak height velocity.

### Anthropometric Measurements

All measurements were taken in the morning by the same experienced researcher. Dimensions included stature, body mass and sitting height. Stretch stature was measured with a wall-mounted stadiometer (±0.1 cm, Holtain Ltd, Crosswell, UK), sitting height with a stadiometer mounted on a purpose-built table (±0.1 cm, Holtain Ltd), body mass with a weighing device (+0.1 kg). The intraclass correlation coefficient for test-retest reliability and typical error of measurement for height, body mass and sitting height were 0.99, 0.98, 0.99, and 0.17%, 0.75% and 0.21%, respectively.

### Illinois Change of Direction Test

**Figure 1:**
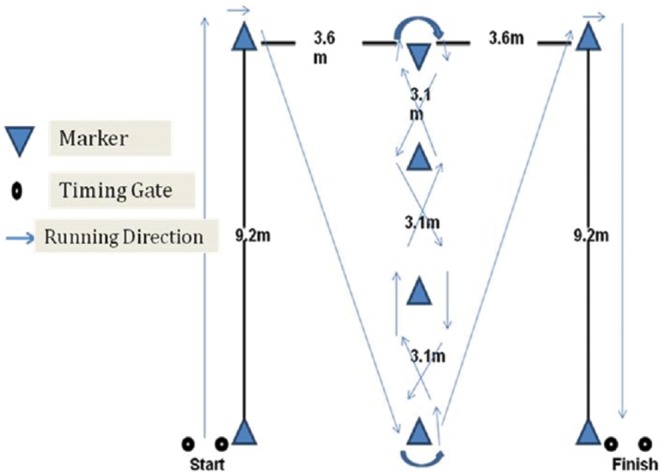
Layout of the Illinois Change of Direction Speed Test (ICODT).

The ICODT test outcomes were recorded using an electronic timing system (Microgate SARL, Italy). Two pairs of the electronic timing system sensors mounted on tripods were set at 1 m above the floor and were positioned 3 m apart facing each other on either side of the starting and finishing lines. To avoid undue switch-on of the timing system, participants had to position the front foot immediately before a line set 0.20 m from the photocell beam. The Illinois CODS test (Figure 1) is set up with four cones forming the agility area. On command, from a standing position athlete sprints 9.2 m, turns and returns back to the starting line, then, he swerves in and out of four markers, completing two 9.2 m sprints to finish the agility course [17]. No technical advice was given as to the most effective movement technique. Athletes were instructed to complete the test as quickly as possible. They were instructed not to cut over the markers but to run around them. If a subject failed to do this, the trial was stopped and re-attempted after the requisite recovery period.

### The Modified Illinois Change of Direction Test

**Figure 2:**
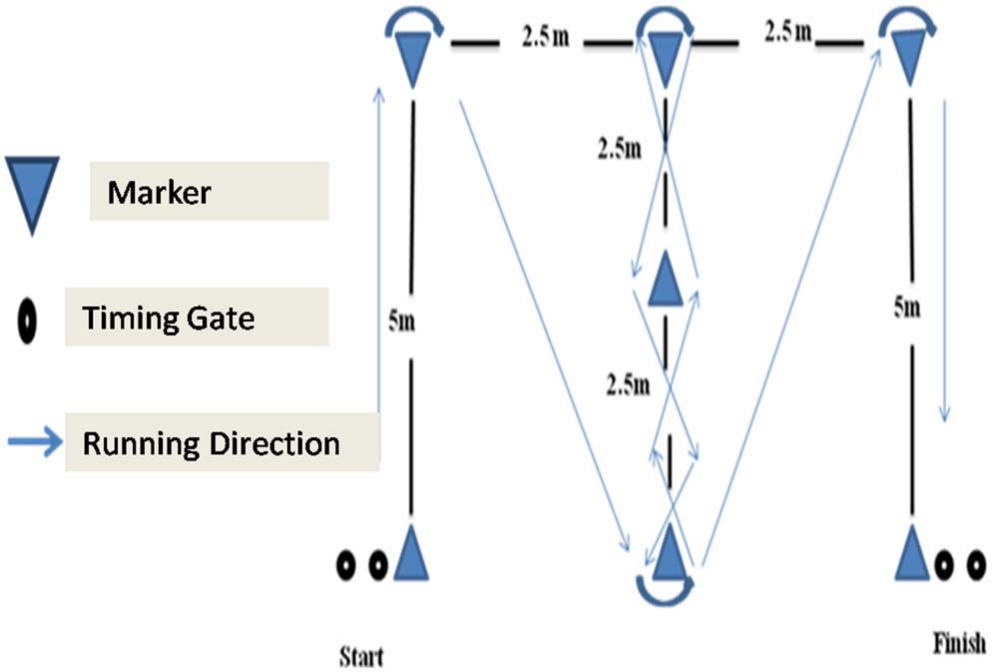
Layout of the Modified Illinois Change of Direction Speed Test (MICODT).

The protocol for the MICODT (Figure 2) was the same as of the Illinois CODS test. Only the total distance covered, measures of inter-cones distance, and the number of the cones was modified. Criteria for accepted test trials were the same as those used on the Illinois CODS test.

### Statistical Analysis

The statistical analyses were carried out using SPSS 19.0 program for Windows (SPSS, Inc, Chicago, IL, USA). Descriptive statistics were generated for all variables. The significance level considered in the present study was set at p<0.05. The normality assumption was checked using Shapiro-Wilk test and all variables in the two phases of the study showed a normal distribution.

Reliability was verified by calculating both intraclass correlation coefficient (ICC) and standard error of measurement (SEM) and construct validity of the MICODT by the receiver operator characteristic (ROC) curve.

Relative reliability was determined by calculating ICC [20]. We considered an ICC below 0.40 as poor, between 0.40 and <0.70 as fair, between 0.70 and <0.90 as good and ≥0.90 as excellent [21]. To test absolute reliability, SEM expressed as coefficient of variation (CV) was calculated. Heteroscedasticity was revealed by calculating the correlation coefficient between the absolute difference and the average of the test trials. The SEM was calculated by dividing the standard deviation of the difference between scores by √2 [20]. As a complement to SEM, the smallest worthwhile change (SWC) was also used. The SWC is determined by the rearrangement of Cohen’s *d* effect size calculation, where the smallest worthwhile effect (0.2) is multiplied by the between-subject SD. By comparing SWC with SEM, test usefulness can be determined using the thresholds proposed by Liow and Hopkins [22]. When SEM ≤ SWC the test’s capacity to detect change is considered “good”; when SEM = SWC it is considered “satisfactory”, and when SEM ≥ SWC the test is rated as “marginal” [20]. Effect size (***d_z_***) was calculated using GPOWER software (Bonn FRG, Bonn University, Department of Psychology) [23]. The following scale was used for the interpretation of ***d_z_***: <0.2, [trivial]; 0.2–0.6, [small]; 0.6–1.2, [moderate]; 1.2–2.0, [large]; and >2.0, [very large] [14], [24]. Pairwise comparisons were applied to determine any learning effect or systematic bias between sample mean scores for test and retest using a paired *t* test. Minimal detectable change (MDC_95%_), which indicate the smallest change that is not due to error, was calculated as MDC_95_ = SEM x√2×1.96 [15].

The criterion related validity has been established by assessing the relationship between MICODT and ICODT using a Pearson product moment correlation coefficient. Vincent [25] has suggested that an absolute correlation coefficient of 0.5–0.7 is considered low, one of 0.7–0.8 is considered moderate, and one of 0.9 or above is considered high.

Unpaired Student t test was used to compare EG and SEG players’ performances in the agility tests. The discriminant ability of the MICODT was analysed using ROC curve. The ability of the new agility test to discriminate young soccer player of different competitive levels was assessed using the area under the ROC curve (AUC) [26], [27]. The ROC curve is a method of assessing the discriminating level of a test used to classify individuals into two groups. It is calculated plotting the sensitivity against 1- specificity, where sensitivity is the percentage of individuals correctly identified by the test (e.g., elite level) and specificity is the percentage of individuals who were sub-elite level and correctly identified by the test. The area under the ROC curve was interpreted as the probability of correctly discriminating between soccer players of different competitive levels. An AUC of 0.5 is interpreted as no discriminatory accuracy and 1.0 as perfect discrimination [27]. As a general rule, the area under the ROC curve >0.70 with a confidence intervals (CI) >0.50 are commonly considered to indicate acceptable discriminative ability [27].

## Results

### Performances on the ICODT and MICODT Tests and Retests

In the analysis of reliability of the ICODT and MICODT, residual data for the two trials were normally distributed (p values ranged from 0.21 to 0.83). The test of equality of means (dependent ***t*** test), showed no significant bias between ICODT and MICODT test and retest. The estimated effect size (***d_z_***), however, was interpreted as being more than trivial for all outcomes for the two CODS tests (Table 1).

**Table 1:** Descriptive statistics and results of relative and absolute reliabilities and MDC_95_% of ICODT and MICODT in U-14 soccer players.

	Trial 1	Trial 2	p	(*d_z_*)	ICC	SEM	SEM as CV	SWC	MDC_95_
	(Mean ± SD)	(Mean ± SD)				(s)	(%)	(s)	(s)
ICODT	18.62±1.26 s	18.60±1.33 s	0.70	0.01	0.94	0.23	1.24	0.25	0.64
MICODT	12.39±1.6 s s	12.38±1.63 s	0.64	0.006	0.99	0.10	0.81	0.33	0.28

*SD: standard deviation;* ***d_z_*** *: effect size; ICODT: Illinois Change of Direction Test; MICODT: Modified Illinois Change of Direction Test; ICC: Intraclass Correlation Coefficient; SEM: Standard Error of Measurement; CV: Coefficient of variation; SWC: smallest worthwhile change; MDC_95_: Minimal Detectable Change.*

### Reproducibility of Performance Scores on the ICODT and MICODT Tests

Both relative and absolute retest reliability of the ICODT and MICODT was excellent (Table 1). The SEM expressed as CV of ICODT and MICODT were within 1%. The ability of the ICODT to detect small performance change was satisfactory, while it was good for the MICODT (Table 1).

### Minimal Detectable Change of ICODT and MICODT Tests

The MDC_95_ for ICODT and MICODT performances are presented in Table 1. The MICODT presents the smallest MDC_95_ value. The Pearson product moment correlation between ICODT and MICODT revealed a very good correlation (r = 0.77; p<0.001).

### Construct Validity of Performance Scores on the ICODT and MICODT Tests

The ICODT and MICODT performances of EG and SEG players are listed in Table 2. Independent sample *t*-test revealed that EG players had significantly better performances (p = 0.005) than SEG players for MICODT. However, the difference observed in these two groups for ICODT was not statistically significant (p = 0.14), showing poor discriminant ability of the ICODT in U-14 soccer players.

**Table 2:** ICODT and MICODT score performances within elite and subelite U-14 soccer players.

Test	Elite player	Sub-elite player	p	(*d_z_*)
ICODT	19.77±0.85 s	20.16±0.70 s	0.14	0.50 [small]
MICODT	12.86 0.41 s	13.32 0.49 s	0.005	1.01 [large]

*ICODT: Illinois Change of Direction Test; MICODT: Modified Illinois Change of Direction Test; dz: Effect size.*

**Figure 3:**
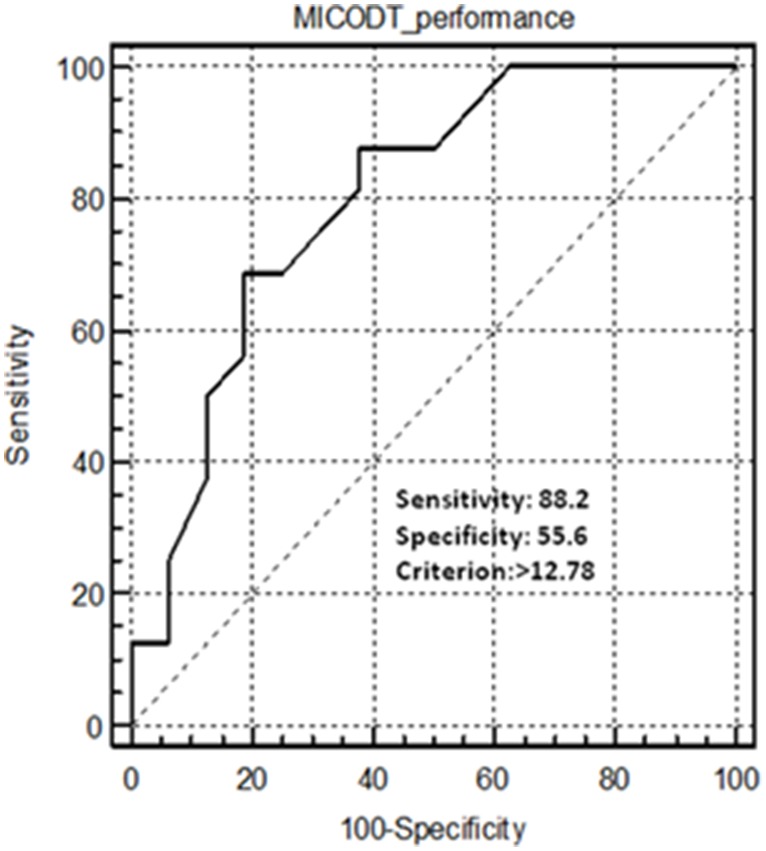
Receiver operating characteristics (ROC) curve for the MICODT between elite and sub-elite U-14 soccer players.

The results of the ROC curve analysis was established between EG and SEG U-14 soccer players. The MICODT was considered to have acceptable discriminant ability. The area under the ROC curve was 0.77 (CI 95%: 0.59 to 0.89, p<0.008) (Figure 3).

## Discussion

To the authors’ knowledge, this is the first study to analyze the reliability and discriminant validity of a new test of change of direction speed (CODS) in U-14 soccer players. The main finding of this study showed that the MICODT is a more reliable and valid test than the ICODT for assessing CODS in U-14 soccer players.

The reliability of fitness testing is a critical issue. It is relevant in establishing the reproducibility of a test and is a prerequisite to establishing validity in future studies. It has been established that within the current investigation, the relative reliability of performance scores of both ICODT and MICODT was excellent. The ICC across the two trials exceeded 0.90. This is consistent with a recent research by Lockie et al. [28] who found a good relative reliability of the ICODT within a sample of soccer players’ athletes. Additionally, these values were comparable to previously reported results on the relative reliability of other agility tests. In this context, Pauole et al. [7] reported an ICC of 0.98 across three CODS T-test trials in college-aged men and women. Sheppard and Young [29] reported an ICC of 0.87 across two reactive agility test trials in thirty-eight Australian football players. Haj-Sassi et al. [30] revealed an ICC of 0.95 in fifty-two male physical education students in a modified CODS T-test.

Absolute reliability was gathered from repeated performances of both tests (i.e., test-retest) by calculating the SEM. A small SEM expressed as CV has been established for ICODT (1.24%) and MICODT (0.81%) showing good absolute reliability of both tests. Lockie et al. [28] revealed that ICODT presented and SEM of 0.29 s (2.5%), supporting the present results. In this study, the likelihood that the true values of the estimated difference in ICODT and MICODT performances (i.e., larger than the SWC) was verified. Results presented in table 1 showed that MICODT had a better ability to detect small performance change in the U-14 soccer players than the ICODT. Its SEM (0.10 s) was smaller than its SWC (0.33 s), while the capacity of the ICODT to detect small performance changes in CODS within U-14 soccer players can only be described as “satisfactory” considering that its SWC (0.25 s) was approximately equal to its SEM (0.23 s). Lockie et al. [28] reported that the SEM of the ICODT was greater than the SWC which means that the usefulness of this test was rated as “marginal”. These findings showed that the usefulness of the ICODT is a subject of discussion.

The SEM allows the calculation of the MDC_95%_. According to Lexell and Downham [31] the MDC_95%_ allows the determination of whether a real change has occurred between test and retest and when a change in a test-retest performance is ±MDC_95%_ a true change is indicated. The inspection of the results presented in Table 1 showed significant differences between the ICODT’s and MICODT’s MDC_95%_ values. The MICODT’s MDC_95%_ (0.28 s) was about half the ICODT’s MDC_95%_ (0.64 s) indicating that the MICODT represents a better ability to detect true changes in CODS than the ICODT in U-14 soccer players.

Pearson product moment correlation coefficients were performed between ICODT and MICODT. The MICODT was significantly associated with the ICODT. The coefficients of determination showed that the MICODT and ICODT shared 60% common variance in U-14 soccer players. These results indicate that the MICODT could be used to evaluate CODS. Even if there is no widely recognised gold-reference agility test, this strong correlation could provide criterion validity to the MICODT as the ICODT has been widely used as an agility test in soccer players.

One of the prerequisites of test applicability in sport science is its construct validity. Construct validity is commonly established by testing differences between groups of subjects of different competitive level [32]. In the research design of the second phase of this study we operated control over construct (i.e., competitive level) supporting variables testing two groups of U-14 soccer players of different competitive level (i.e., EG and SEG). Results showed significant differences in performances scores in the MICODT between the two groups (i.e., EG vs. SEG) (Table 2). The presence of competitive-level sensitivity of the MICODT was further supported by ROC analysis. Recently, this statistical test has gained popularity [13]. It is currently used to study the discriminant validity of physiological and performance testing [27], [32]. Thus, a test was considered as discriminant if its area under the ROC curve was equal to or greater than 0.70 [26], [27]. In the present study, the area under the ROC curve represented the probability of correctly discriminating elite from sub-elite U-14 soccer player using the MICODT. Results showed that the area under the ROC curve was above 0.70 (0.77; CI 95%: 0.59–0.89) and that the test score able to distinguish between the two different competitive levels group was 12.78 s. This cut-off value gives a “true-positive rate” (sensitivity) of 88.2% and a “false-positive rate” (1-specificity) of 55.6% (Figure 3). Thus, ROC curve analysis suggests that the MICODT has acceptable discriminant ability if used to differentiate between elite and sub-elite U-14 soccer players. Additionally, such a test can guide coaches more effectively through conditioning and training programs across a season and also can help coaches to better understand differences between players in talent selection and identification. These results are consistent with previous findings within rugby players [33]. Authors revealed that the academy group was significantly faster in CODS performance compared to a club level group. Another study by Farrow et al. [34] reported that highly-skilled net ball players group was faster than the lesser-skilled ones in CODS performance. However, this kind of discriminative ability is absent for the ICODT, in view of the fact that no significant difference was detected between mean performances relative to the ICODT across U-14 soccer players’ levels of expertise (Table 2).

Overall, it seems that by keeping the same nature of displacement and reducing the distance to be covered (i.e., from 60 m to ∼30 m) and by modifying the starting position (i.e., from a prone position to a standing one) during the novel proposed CODS test (i.e., MICODT) make it more specific and less strenuous within U-14 soccer players when compared to the old version. This is supported by the better relative and absolute reliability as well as validity results of the MICODT compared to ICODT in U-14 soccer players.

## Conclusion

Results from the present investigation suggest that the MICODT is a reliable and valid CODS test that can discriminate between U-14 soccer players of varying playing abilities. The use of the MICODT can be favoured instead of the ICODT since it could provide an alternative, more valid assessment of CODS. It can be used regularly, because of its simplicity and low cost, to monitor training programs intervention directed toward improving CODS performance within U-14 soccer players. Additionally, from the results presented above it seems that the MICODT has a better ability to detect true change in agility performance when compared to ICODT. This characteristic of the MICODT is of utmost importance to monitor accurately agility’s performance level of U-14 soccer players across the time. However, the main limitation of the current investigation is that the new test (MIOCDT), as with the old version (IOCDT), only assesses the physical component of agility (i.e., CODS). As cognitive and perceptual factors contribute a lot to agility performance, future studies testing both the physical and perceptual (i.e., decision making) components of this sports’ critical task (i.e. agility) in young soccer athletes must be conducted. It should also be noted, that in view of the low sample size for the ROC analysis, further studies, on a larger sample, should be conducted to confirm the present findings.

## References

[1] Davids K, Lees A, Burwitz L (2000) Understanding and measuring coordination and control in kicking skills in soccer. Implications for talent identification and skill acquisition. *J Sports Sci* 18: 703–714. 11043896 10.1080/02640410050120087

[2] Harman E, Rosenstein M, Frykman P, Rosenstein R (1990) The effects of arm and counter-movement on vertical jumping. *Med Sci Sports Exerc* 22: 825–833. 2287261 10.1249/00005768-199012000-00015

[3] Reilly T, Bangsbo J, Franks A (2000) Anthropometric and physiological predispositions for elite soccer. *J Sports Sci* 18: 669–683. 11043893 10.1080/02640410050120050

[4] Sheppard JM, Young WB, Doylec TLA, Sheppard TA, Newton RU (2006a) An evaluation of a new test of reactive agility and its relationship to sprint speed and change of direction speed. *J Sci Med Sports* 9: 342–349. 10.1016/j.jsams.2006.05.019 16844413

[5] Bloomfield JTR, Polman R, O’Donoghue P (2007) Physical demands of different positions in FA premier league soccer. *J Sports Sci Med* 6: 63–70. 24149226 PMC3778701

[6] Haj-Sassi R, Dardouri W, Gharbi Z, Chaouachi A, Mansour H, et al (2011) Reliability and validity of a new repeated agility test as a measure of anaerobic and explosive power. *J Strength Cond Res* 25: 472–480. 21240028 10.1519/JSC.0b013e3182018186

[7] Pauole KK, Madole J, Garhammer M, Lacourse M, Rozenek R (2000) Reliability and validity of the T-test as a measure of agility, leg power, and leg speed in college-aged men and women. *J Strength Cond Res* 14: 443–450.

[8] Hachana Y, Chaabène H, Nabli MA, Attia A, Moualhi J (2013) et al Test-retest reliability, criterion-related validity, and minimal detectable change of the Illinois agility test in male team sport athletes. *J Strength Cond Res* . 27(10): 2752–9. 10.1519/JSC.0b013e3182890ac3 23439329

[9] Draper JA, Lancaster MG (1985) The 505 test: A test for agility in the horizontal plane. *Aust J Sci Med Sports* 17(1): 15–18.

[10] Meir R, Newton R, Curtis E, Fardell M, Butler B (2001) Physical fitness qualities of professional rugby league football players: Determination of positional differences. *J Strength Cond Res* 15: 450–458. 11726256

[11] Little T, Williams AG (2005) Specificity of acceleration, maximum speed, and agility in professional soccer players. *J Strength Cond Res* 19: 76–78. 15705049 10.1519/14253.1

[12] Brughelli M, Cronin J, Levin G, Chaouachi A (2008) Understanding change of direction ability in sport. *Sports Med* 38: 1045–1063. 19026020 10.2165/00007256-200838120-00007

[13] Caldwell BP, Peters DM (2009) Seasonal variation in physiological fitness of a semiprofessional soccer team. *J Strength Cond Res* 23: 1370–1377. 19620929 10.1519/JSC.0b013e3181a4e82f

[14] Hastad DN, Lacy AC (1994) Measurement and evaluation in physical education and exercise science (2nd edn.). Scottsdale, AZ: GorsuchScarisbric.

[15] Jarvis S, Sullivan LO, Davies B, Wiltshire H, Baker JS (2009) Interrelationships between measured running intensities and agility performance in subelite rugby union players. *Res Sports Med* 17: 217–30. 19967601 10.1080/15438620903323892

[16] Stewart PF, Turner AN, Miller SC (2012) Reliability, factorial validity, and interrelationships of five commonly used change of direction speed tests. *Scand J Med Sci Sports*, [Epub ahead of print]. 10.1111/sms.12019 23176602

[17] Vescovi JD, McGuigan MR (2008) Relationships between sprinting, agility, and jump ability in female athletes. *J Sports Sci* 26: 97–107. 17852692 10.1080/02640410701348644

[18] Castagna C, D’Ottavio S, Abt G (2003) Activity profile of young soccer players during actual match play. *J Strength Cond Res* 17: 775–780. 14636107 10.1519/1533-4287(2003)017<0775:apoysp>2.0.co;2

[19] Mirwald RL, Baxter-Jones AD, Bailey DA, Beunen GP (2002) An assessment of maturity from anthropometric measurements. *Med Sci Sports Exerc* 34: 689–694. 11932580 10.1097/00005768-200204000-00020

[20] Hopkins WG (2000) Measures of reliability in sports medicine and science. *Sports Med* 30: 1–15. 10907753 10.2165/00007256-200030010-00001

[21] Coppieters M, Stappaerts K, Janssens K, Jull G (2002) Reliability of detecting onset of pain and sub-maximal pain during neural provocation testing of the upper quadrant. *Physiother Res Int* 7: 146–156. 12426912 10.1002/pri.251

[22] Liow DK, Hopkins WG (2003) Velocity specificity of weight training for kayak sprint performance. *Med Sci Sports Exerc* 35: 1232–1237. 12840647 10.1249/01.MSS.0000074450.97188.CF

[23] Faul F, Erdfelder E (2004) GPOWER. A Priori, Post-Hoc, and Compromise Power Analyses for MS-DOS (Computer Program). Bonn, FRG: Bonn University, Department of Psychology.

[24] Scanlan AT, Dascombe BJ, Reaburn PRJ (2012) The construct and longitudinal validity of the basketball exercise simulation test. *J Strength Cond Res* 26: 523–530. 22240548 10.1519/JSC.0b013e318220dfc0

[25] Vincent WJ (1999) Statistics in Kinesiology (2nd edition). Champaign, IL: Human Kinetics.

[26] Impellizzeri FM, Marcora SM (2009) Test Validation in Sport Physiology: Lessons Learned From Clinimetrics. *Inter J Sports Physiol Perf* 4: 269–277. 10.1123/ijspp.4.2.269 19567929

[27] Menespa P, Aldo S, Impellizzeri FM (2010) Aerobic Fitness Variables Do Not Predict the Professional Career of Young Cyclists. *Med Sci Sports Exerc* 42: 805–812. 19952851 10.1249/MSS.0b013e3181ba99bc

[28] Lockie RG, Schultz AB, Callaghan SJ, Jeffriess MD, Berry SP (2013) Reliability and Validity of a New Test of Change-of-Direction Speed for Field- Based Sports: the Change-of-Direction and Acceleration Test (CODAT). *J Sports Sci Med* 12: 88–96. 24149730 PMC3761765

[29] Sheppard JM, Young WB (2006b) Agility literature review: Classifications, training and testing. J Sports Sci 24: 919–932. 16882626 10.1080/02640410500457109

[30] Haj Sassi R, Dardouri W, Haj Yahmed M, Gmada N, Mahfoudi MH, et al (2009) Relative and absolute reliability of a modified agility T-test and its relationship with vertical jump and straight sprint. *J Strength Cond Res* 23: 1644–1651. 19675502 10.1519/JSC.0b013e3181b425d2

[31] Lexell JE, Downham DY (2005) How to assess the reliability of measurements in rehabilitation. *Am J Physical Med Rehabil* 84: 719–23. 10.1097/01.phm.0000176452.17771.20 16141752

[32] Castagna C, Impellizzeri FM, Bizzi M, Weston M, Manzi V (2011) Applicability of a change of direction ability field test in soccer assistant referees. *J Strength Cond Res* 25: 860–866. 21325915 10.1519/JSC.0b013e318208ae8e

[33] Green BS, Blake C, Caulfield BM (2011) A valid field test protocol of linear speed and agility in rugby union. *J Strength Cond Res* 25(5): 1256–62. 21116198 10.1519/JSC.0b013e3181d8598b

[34] Farrow D, Young W, Bruce L (2005) The development of a test of reactive agility for netball: A new methodology. *J Sci Med Sport* 8: 52–60. 15887901 10.1016/s1440-2440(05)80024-6

